# Organobase-catalyzed three-component reactions for the synthesis of 4*H*-2-aminopyrans, condensed pyrans and polysubstituted benzenes

**DOI:** 10.3762/bjoc.10.11

**Published:** 2014-01-14

**Authors:** Moustafa Sherief Moustafa, Saleh Mohammed Al-Mousawi, Maghraby Ali Selim, Ahmed Mohamed Mosallam, Mohamed Hilmy Elnagdi

**Affiliations:** 1 Department of Chemistry, Faculty of Science; University of Kuwait, Safat, 13060, P.O. Box 5969, Kuwait; 2 Department of Chemistry, Faculty of Science at Qena, South Valley University, P.O. Box 83523, Qena, Egypt

**Keywords:** aminopyranes, arylbenzoic acid, DABCO, L-proline, multicomponent, tetrahydronaphthalene, three-component reaction

## Abstract

Novel routes for the preparation of 2-amino-4*H*-pyran-3-carbonitrile **9**, amino-arylbenzoic acid ester derivatives **13a**,**b**, 2-aminotetrahydro-4*H*-chromene-3-carbonitrile **18**, 3-amino-4-cyanotetrahydronaphthalene-2-carboxylic acid ester **26** and 4-amino-3,5-dicyanophthalic acid ester derivatives **37a–c** were developed. The synthetic methods utilize one-pot reactions of acetylene carboxylic acid esters, α,β-unsaturated nitriles and/or active methylenenitriles in the presence of L-proline or DABCO. Plausible mechanisms are suggested for the formation of the products. Finally, these compounds were used for the efficient synthesis of 6-amino-5-cyanonicotinic acid ester derivatives **31a**,**b**, ethyl 4-amino-5*H*-pyrano[2,3-*d*]pyrimidine-6-carboxylates **33a**,**b**, 4-amino-6*H*-pyrrolo[3,4-*g*]quinazoline-9-carbonitrile **39**, and 1,7-diamino-6-(*N*'-hydroxycarbamimidoyl)-3-oxo-5-phenyl-3*H*-isoindole-4-carboxylate (**40**).

## Introduction

**Scheme 1:**
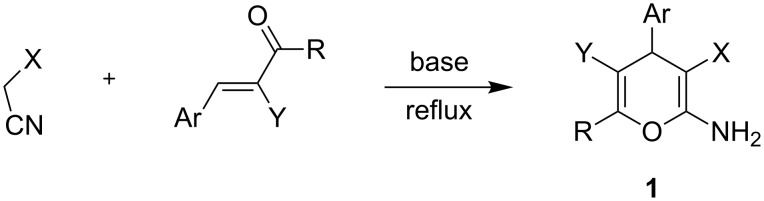
Reaction of active methylenenitriles with α,β-unsaturated ketones **1**.

The reaction of arylidenemalononitriles with active methyl and methylene compounds was extensively utilized for the syntheses of otherwise non-readily obtainable pyrans [1–3], pyridines [3–5] and polysubstituted aromatics [6–7]. The synthesis of 2-amino-4*H-*pyrans by these reactions has recently been surveyed. Since the first report describing the preparation of 2-amino-4*H*-pyrans by the addition of active methylenenitriles to α,β-unsaturated ketones [8], 2-amino-4*H*-pyrans **1** (Scheme 1) have become the central focus of a number of chemical and biological investigations [9–10].

**Scheme 2:**
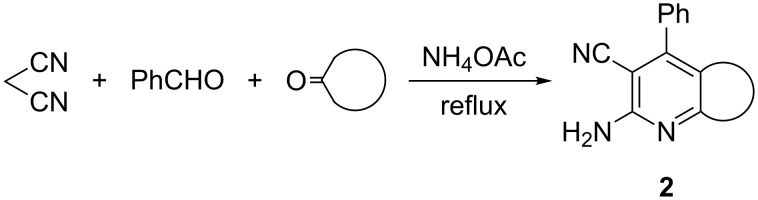
Synthesis of 2-amino-4-phenyl-3-cyanopyridines **2**.

Perez et al. recently reported an interesting method for the synthesis of condensed pyridines **2** through a three-component reaction of malononitrile with benzaldehyde and cyclic ketones [11] (Scheme 2).

**Scheme 3:**
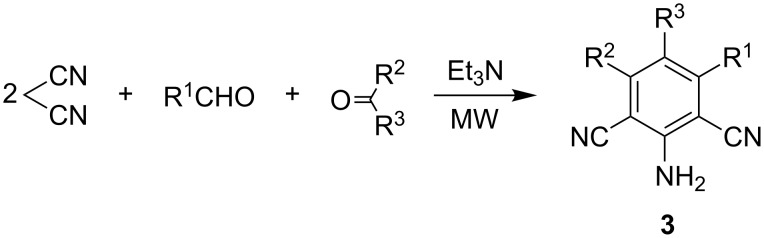
Synthesis of polysubstituted benzenes **3**.

In contrast, MW-irradiation-promoted reactions of ketones, aldehydes and malononitrile are known to afford polysubstituted benzenes **3** (Scheme 3) [12].

Elnagdi et al. reported [13] that reaction of alkyl azenyl carbonitrile with arylidene malononitrile afforded benzo-fused azenes. Based on these studies several surveys were published during the last decade [10,14–19].

## Results and Discussion

**Scheme 4:**
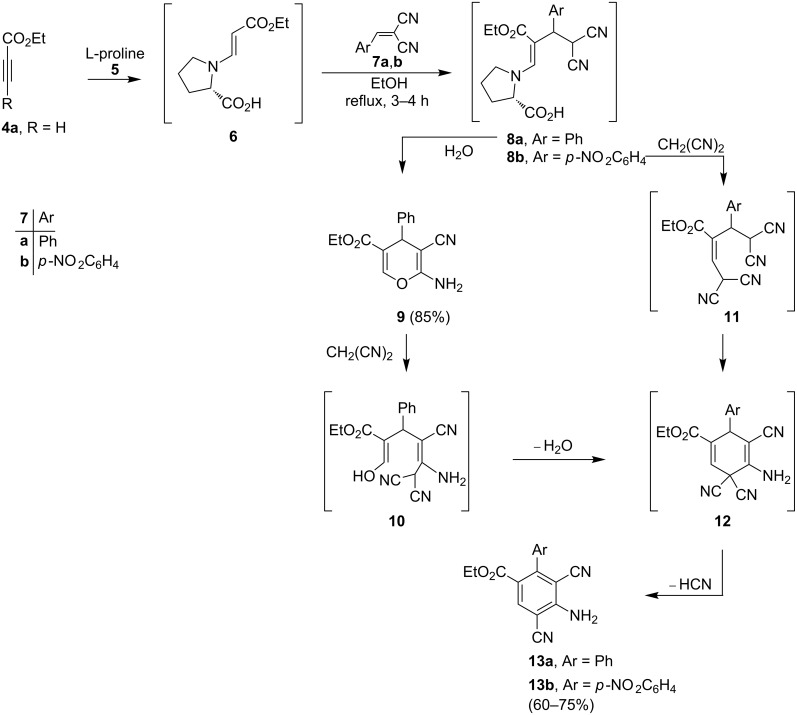
Syntheses of compound **9** and compounds **13a**,**b**.

Very recently we reported that the reaction of dimethylacetylene dicarboxylate (DMADC) with benzylidenemalononitrile afforded 2-amino-4*H*-pyran **9** and that the yield of the process can be improved if equimolar amounts of DMADC, benzylidenemalononitrile and malononitrile are used [20]. Similar observations have been made in previous studies of these reactions using 1-methylimidazole [21]. Recently we observed that 2-amino-4*H*-pyran **9** can be generated using a one-pot reaction involving condensation of ethyl propiolate (**4a**) with benzylidenemalononitrile (**7a**) in the presence of L-proline (**5**) (Scheme 4) [4].

**Figure 1:**
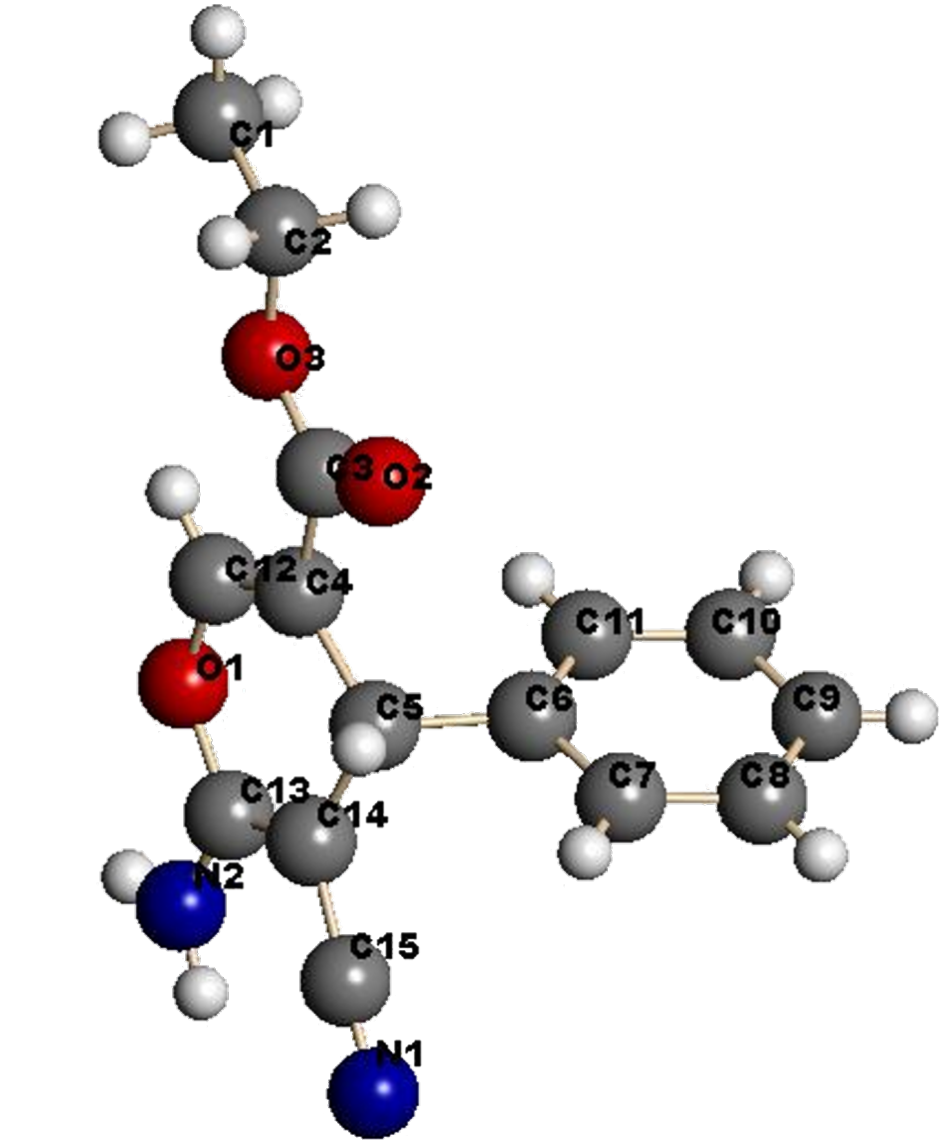
X-ray crystal structure of **9**.

It is believed that the pathway followed in this process involves conjugate addition of proline to the propiolate to yield adduct **6** which then reacts with **7a** to form the enamino ester **8a**. Hydrolysis of **8a** followed by cyclization affords **9** in 85% yield. The structure of **9** was unambiguously assigned by X-ray crystallographic methods (Figure 1).

**Figure 2:**
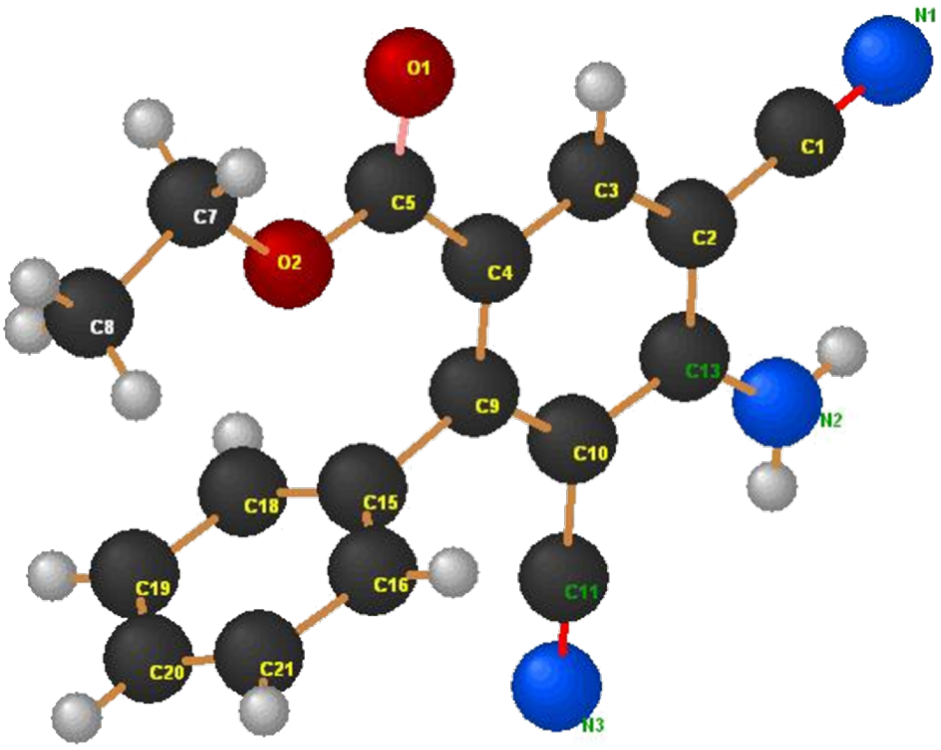
X-ray crystal structure of **13a**.

In contrast, we found that the *p*-nitrophenyl-substituted analogue **7b** reacts with ethyl propiolate (**4a**) in the presence of L-proline to produce the penta-substituted benzene derivative **13b** in 60% yield (Scheme 4). In order to explain this unusual finding, we proposed that malononitrile, perhaps formed under the reaction conditions by C–C bond cleavage promoted fragmentation of **8**, adds to **9** to generate **10** that cyclizes to form **12**. Subsequent aromatization of **12** produces the benzoate derivative **13b**. Alternatively, **8** could react with malononitrile to yield **11** that then serve as a precursor to **13** (Scheme 4). In support of the former mechanistic proposal, it was observed that 2-amino-4*H*-pyran **9** reacts with malononitrile to yield **13a** (Scheme 4), whose structure was unambiguously assigned by X-ray crystallographic analysis (Figure 2).

This finding led to the development of a procedure for the high yielding preparation of **13b** that involves the addition of one equivalent of malononitrile to the reaction mixture containing ethyl propiolate (**4a**) and **7b**.

**Figure 3:**
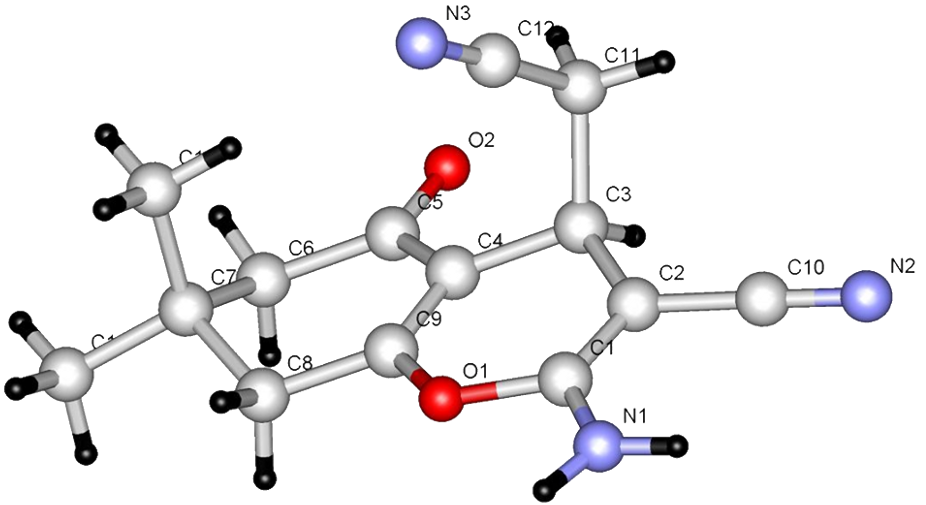
X-ray crystal structure of **18**.

The observations described above prompted us to extend the synthetic potential of these types of condensation reactions. In the following studies, we found that 5,5-dimethylcyclohexane-1,3-dione (**14**) reacts with enaminonitrile **15** and malononitrile in the presence of L-proline or DABCO to yield the 2-amino-4*H*-pyran **18** whose structure was assigned by X-ray crystallographic methods (Figure 3).

**Scheme 5:**
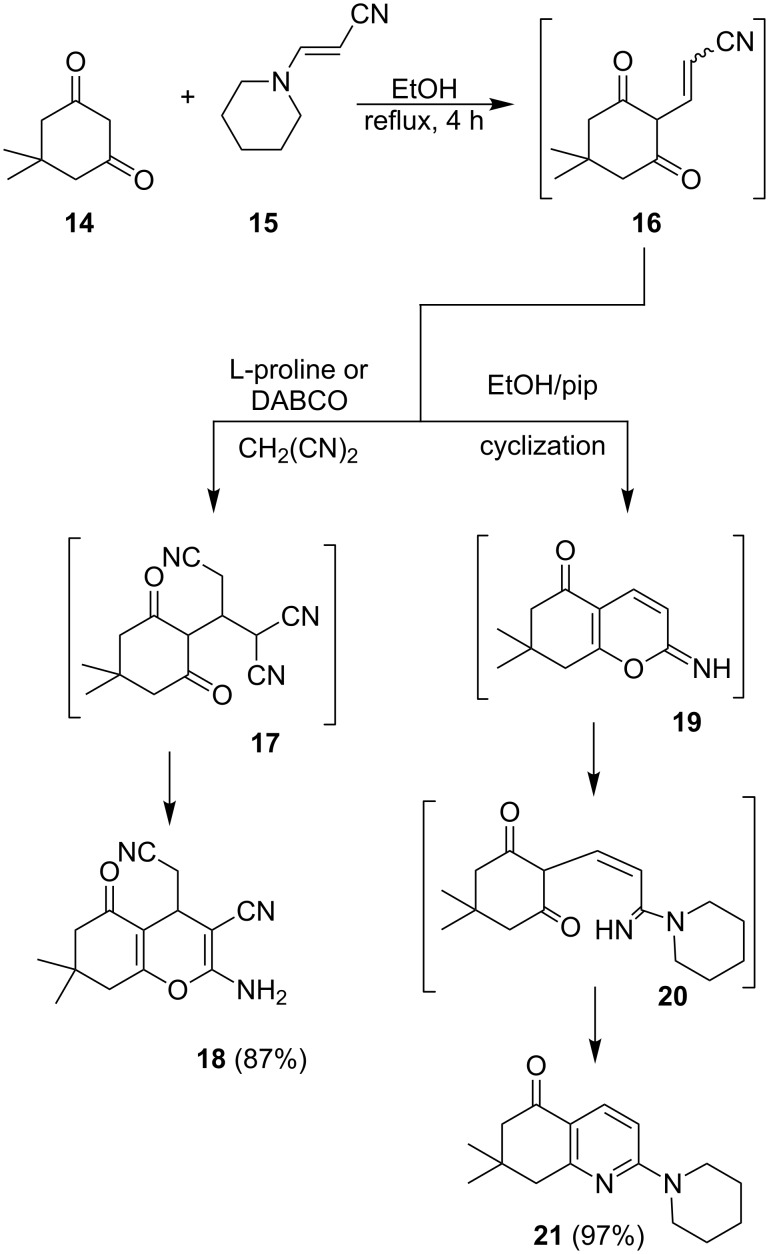
Syntheses of compounds **18** and **21**.

**Figure 4:**
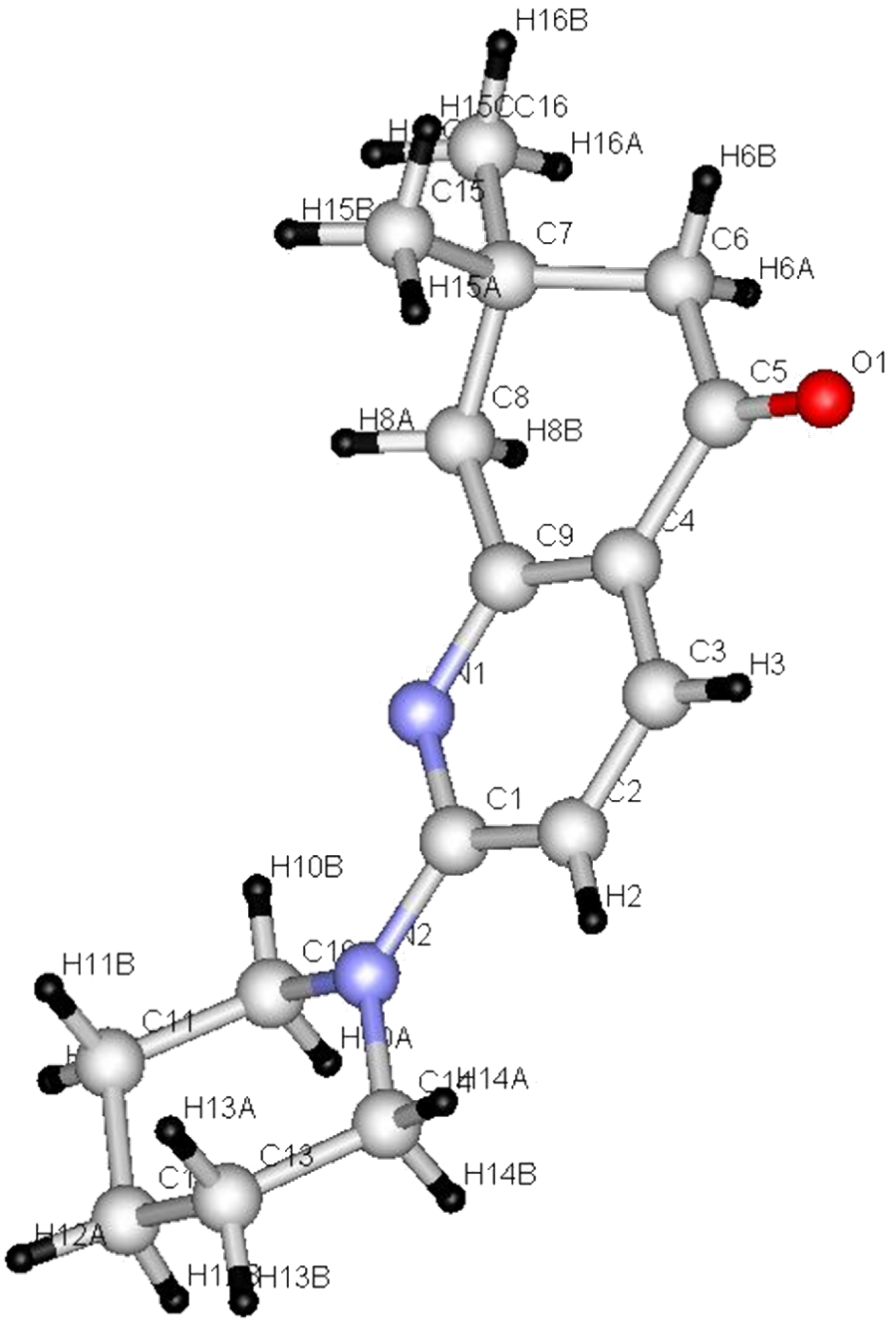
X-ray crystal structure of **21**.

We assumed that in this process **14** and **15** undergo an initial condensation to yield dione **16** that reacts with malononitrile to afford adduct **17**, which subsequently cyclizes to produce **18**. It is of value to note that, when the reaction of **14** and **15** is conducted in the absence of malononitrile, dihydroquinolinone **21** is generated through a pathway involving the intermediate imines **19** and **20** (Scheme 5). The structure of **21** was determined by X-ray crystallographic methods (Figure 4).

**Figure 5:**
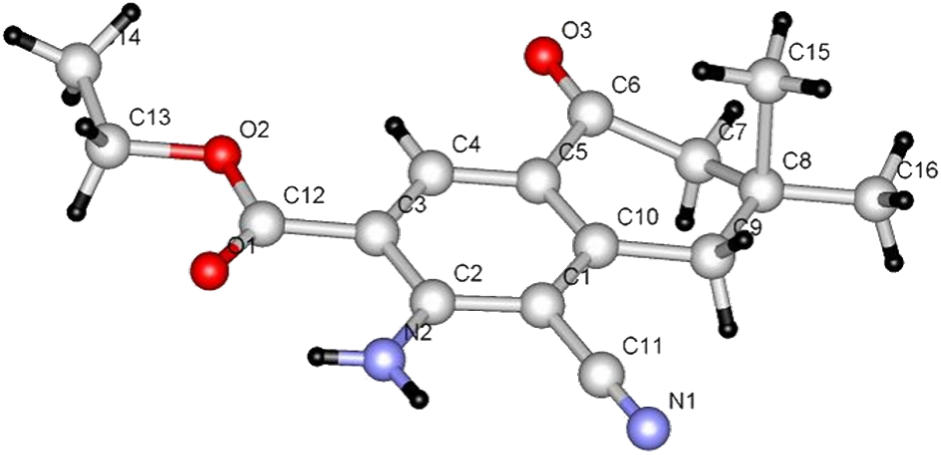
X-ray crystal structure of **26**.

An attempt to prepare the bicyclic 2-amino-4*H*-pyran **24** by the reaction of dione **14** with ethyl propiolate (**4a**) in the presence of malononitrile and L-proline or DABCO was not successful. Instead, these substances reacted to the fused benzoic acid ester **26**, which was characterized by X-ray crystallography (Figure 5).

**Scheme 6:**
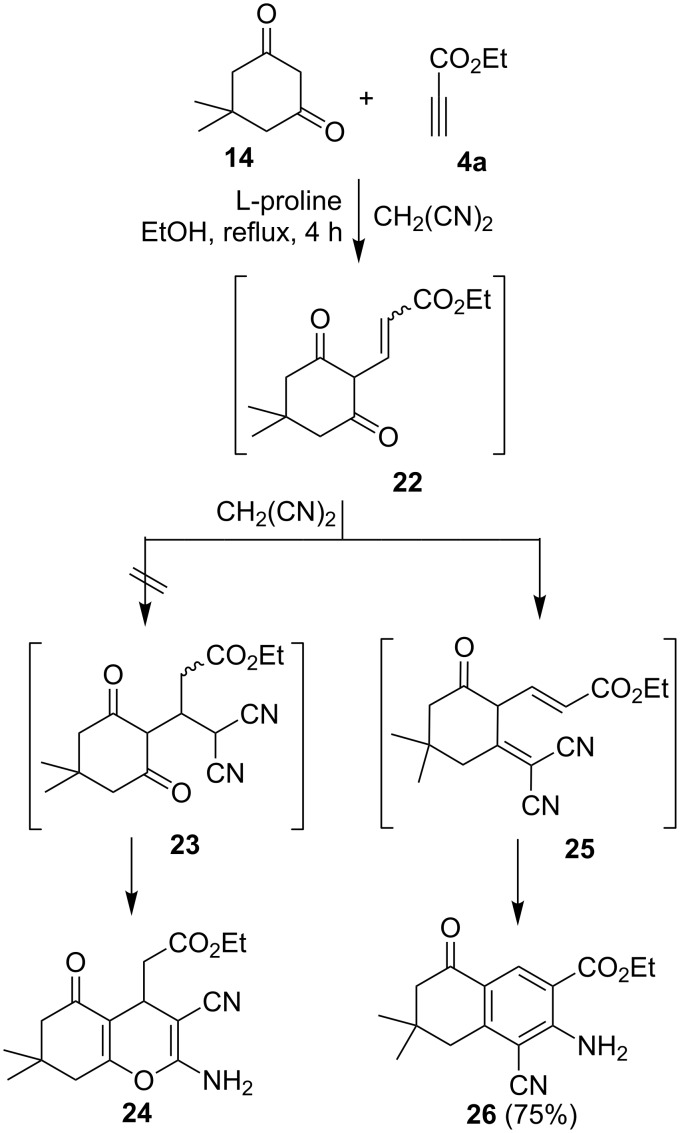
Syntheses of compound **26**.

It appears that in this process, ethyl propiolate (**4a**) reacts with dione **14** initially to form adduct **22** which then adds to malononitrile to produce **25**. The latter undergoes cyclization to afford **26** (Scheme 6).

**Figure 6:**
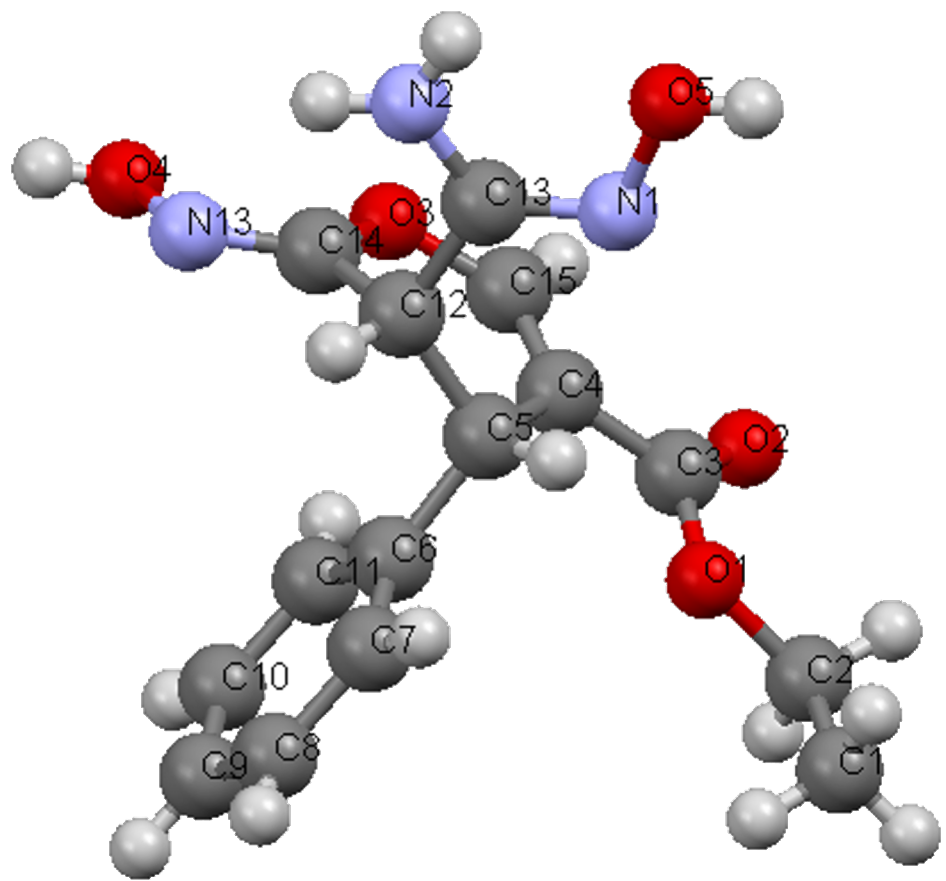
X-ray crystal structure of **27a**.

**Scheme 7:**
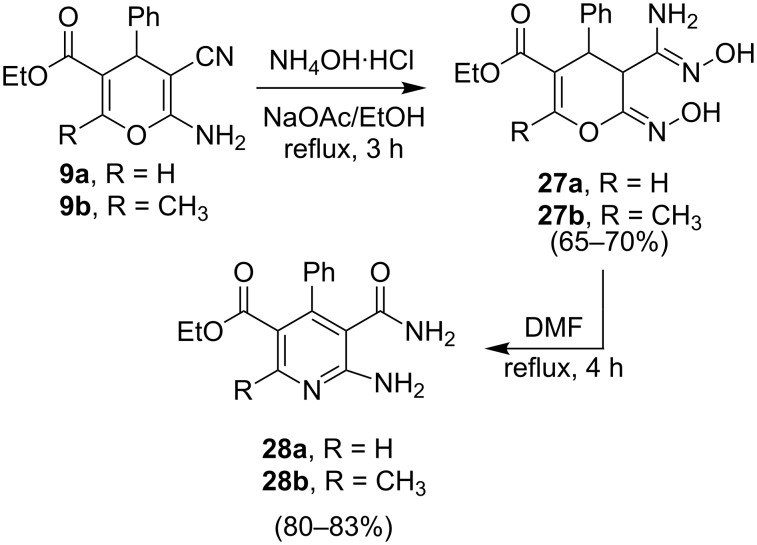
Syntheses of compound **28a**,**b**.

**Figure 7:**
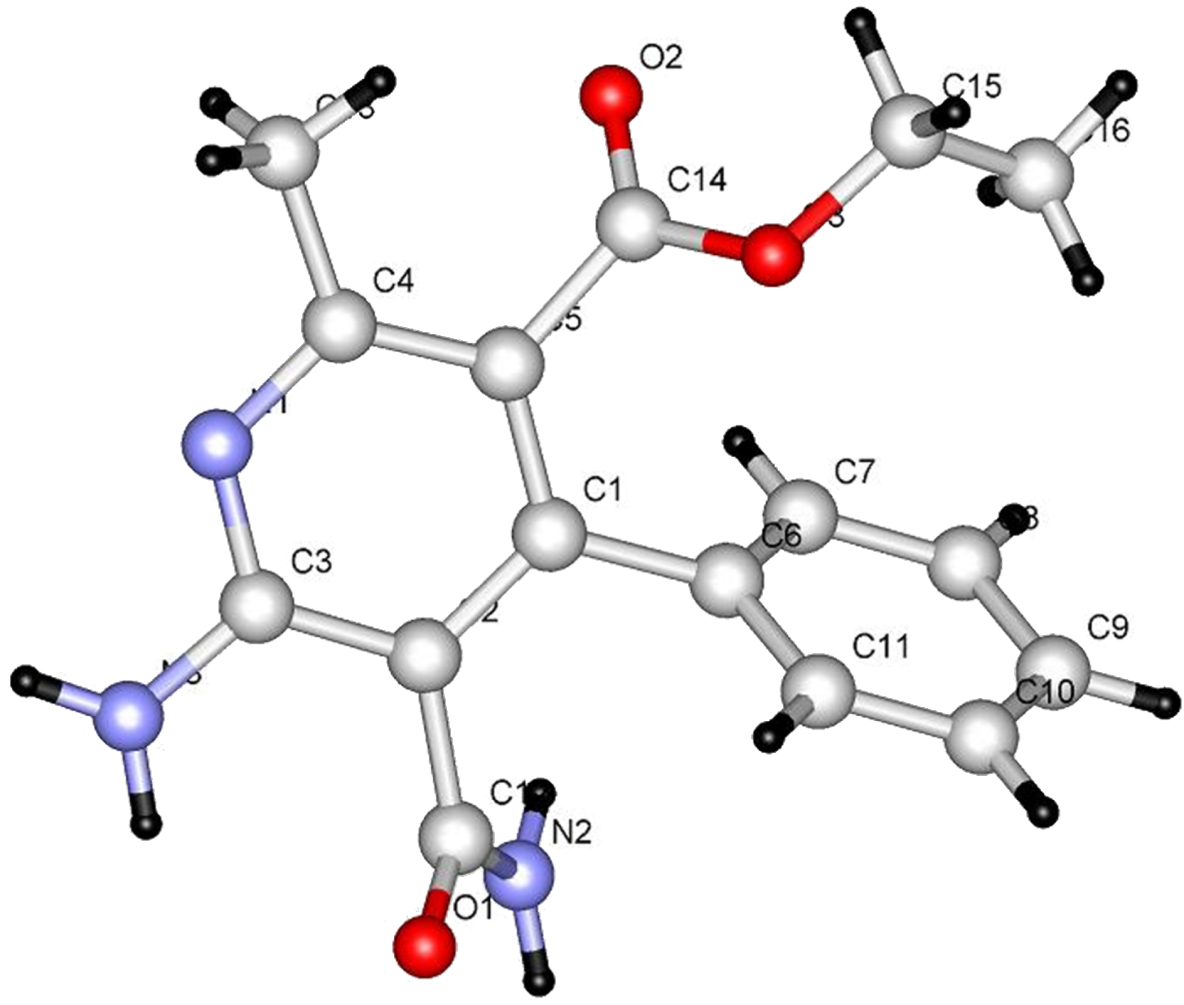
X-ray crystal structure of **28b**.

It is interesting that the 2-amino-4*H*-pyrans, generated in the reactions described above, serve as excellent precursors to uniquely substituted nicotinate derivatives. For example, both pyrans **9a** and **9b** [22] react with hydroxylamine hydrochloride in ethanolic solutions containing sodium acetate to yield the respective amidoximes **27a** and **27b**. The structures of these compounds were assigned by X-ray crystallographic methods (Figure 6). In addition, these substances can be transformed to the corresponding ethyl 6-amino-5-carbamoyl-4-phenylnicotinate derivatives **28a** and **28b** by stirring in refluxing DMF (Scheme 7, Figure 7).

**Figure 8:**
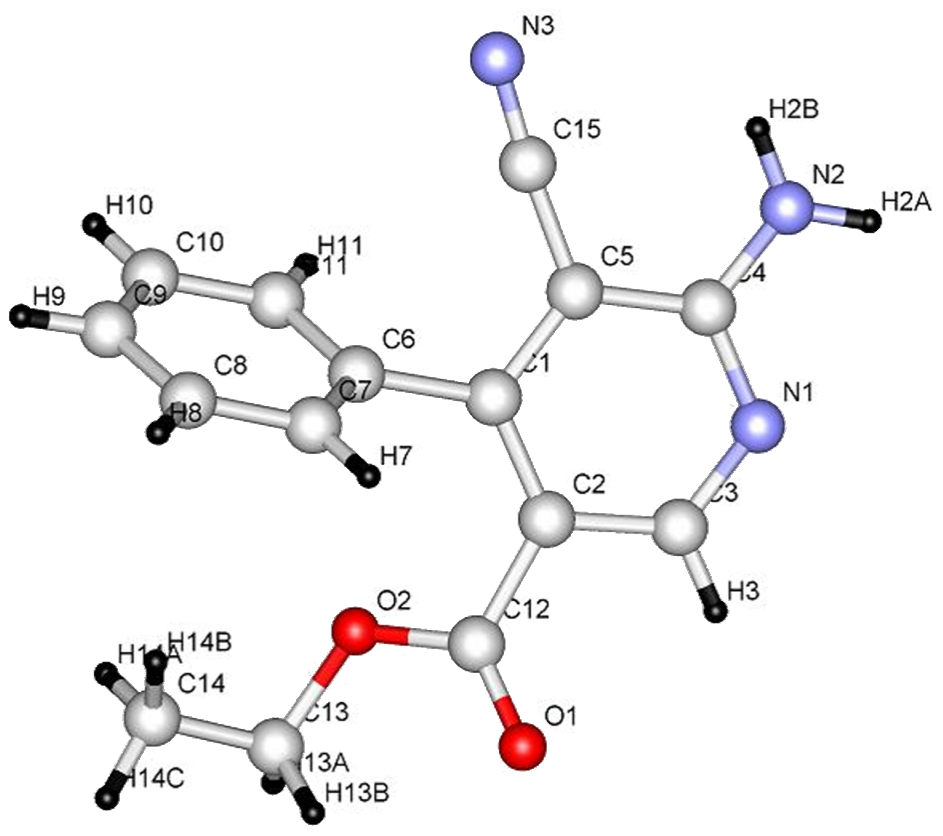
X-ray crystal structure of **31a**.

**Figure 9:**
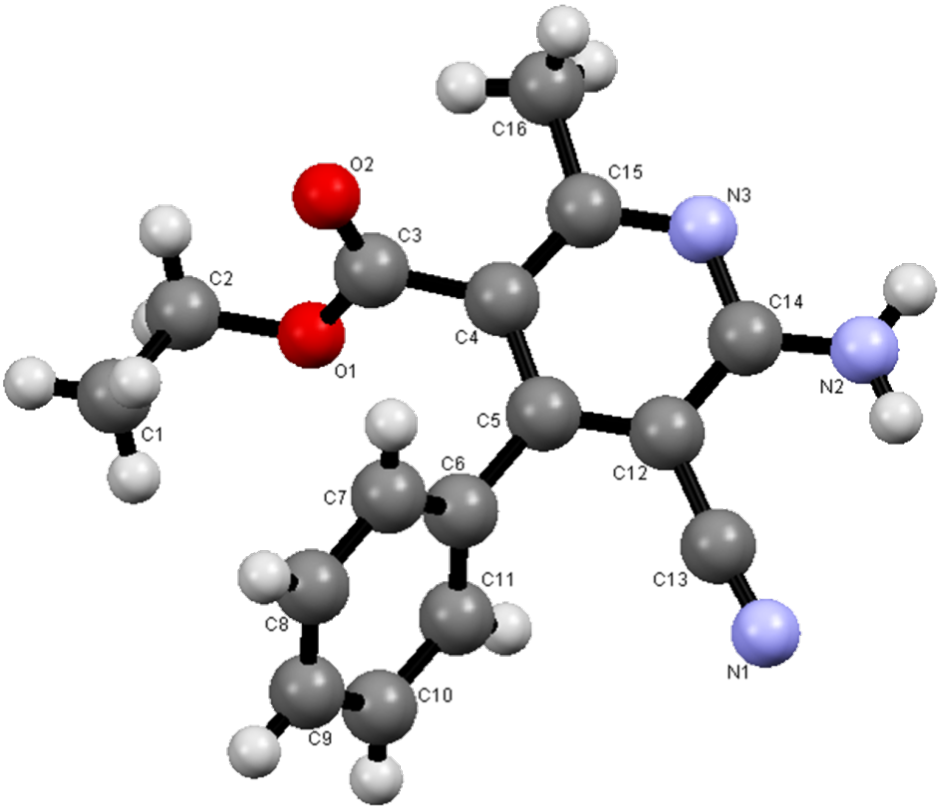
X-ray crystal structure of **31b**.

Furthermore, **9a** and **9b** rearrange to the corresponding nicotinic acid derivatives **31a** and **31b** when they are stirred in refluxing acetic acid containing ammonium acetate. The structures of both of the products were assigned by employing X-ray crystallographic methods (Figure 8 and Figure 9).

**Scheme 8:**
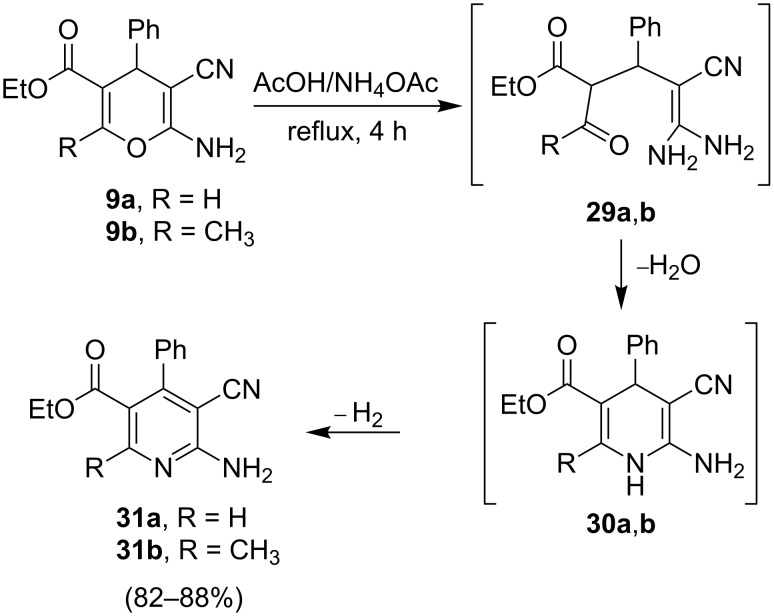
Syntheses of compound **31a**,**b**.

We believe that the nicotinic acid esters are generated in these reactions by ring opening of **9a** and **9b** to yield the respective amidines **29a** and **29b**, which then cyclize to produce **30a** and **30b**. The latter compounds readily undergo autooxidation to form **31a** and **31b** (Scheme 8).

**Scheme 9:**
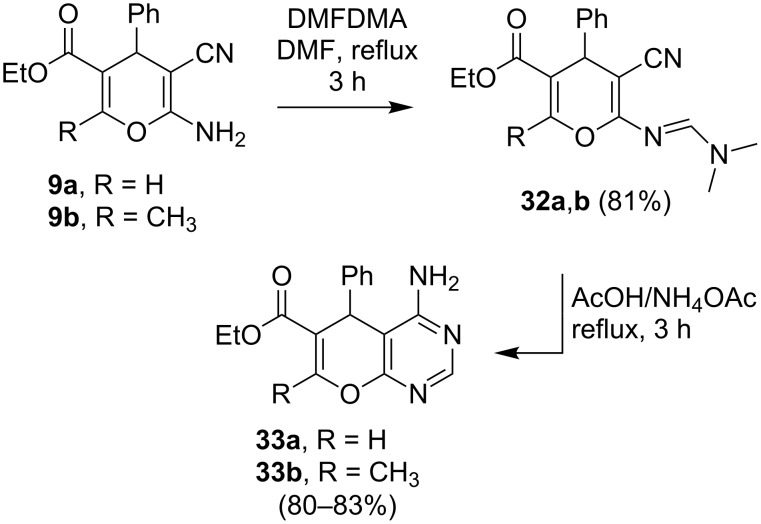
Syntheses of compound **33a**,**b**.

**Figure 10:**
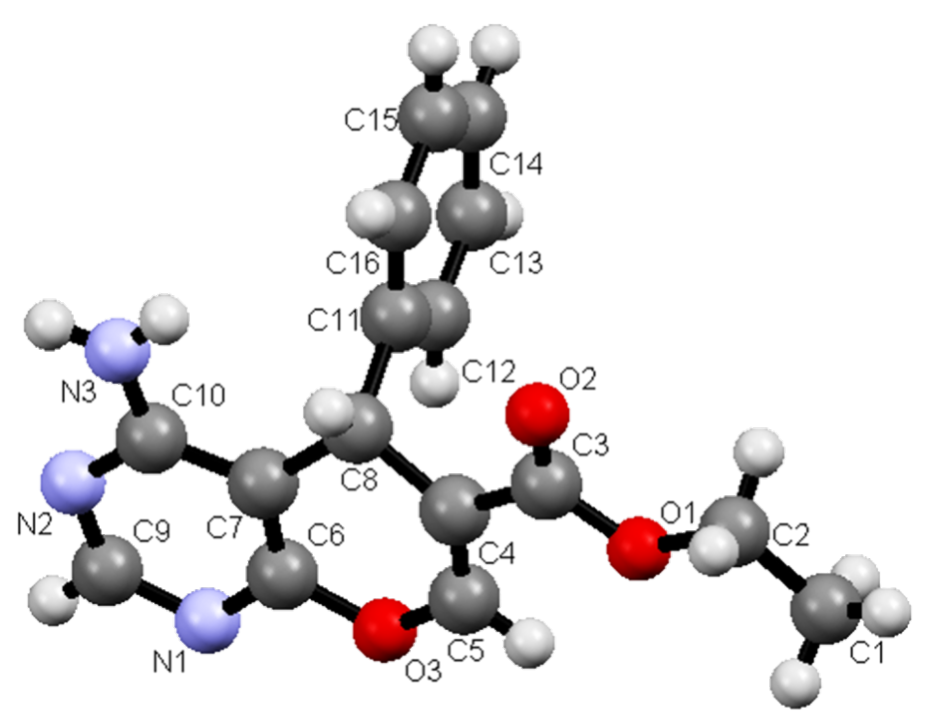
X-ray crystal structure of **33a**.

Finally, the pyranopyrimidines **33a** and **33b** were efficiently produced by a sequence including an initial condensation reaction of the 2-amino-4*H*-pyrans **9a** and **9b** with dimethylformamide dimethylacetal (DMFDMA) to yield the amidine-substituted derivatives **32a** and **32b**. Stirring solutions of these substances in refluxing AcOH/NH_4_OAc gave the respective pyranopyrimidines **33a** and **33b** (Scheme 9, Figure 10).

**Scheme 10:**
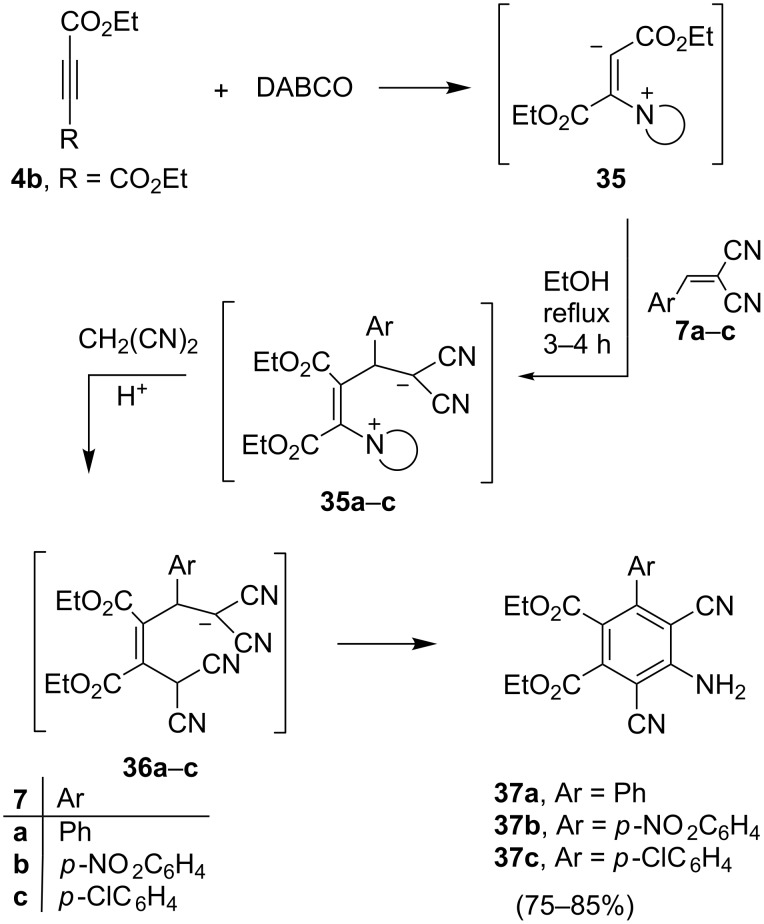
Syntheses of compound **37a**–**c**.

**Figure 11:**
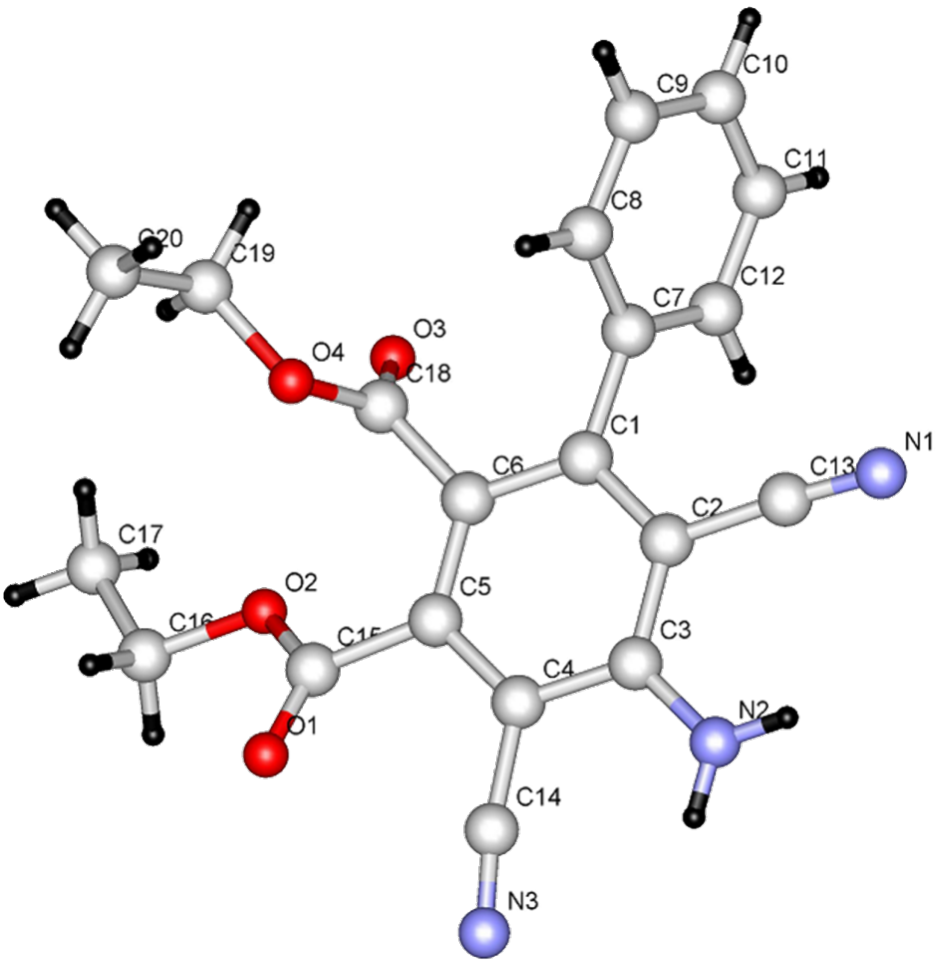
X-ray crystal structure of **37a**.

In an exploratory study aimed at expanding the chemistry shown in Scheme 4, we observed that diethyl acetylenedicarboxylate (DEAD, **4b**) does not react with benzylidenemalononitrile (**7a**) in the presence of L-proline. However, when DABCO was employed as the amine nucleophile, reaction of DEAD with **7a** readily took place and the substituted phthalate diester **37a** was obtained in 80% yield. The structure of **37a** was determined by X-ray crystallographic analysis (Scheme 10 and Figure 11).

**Figure 12:**
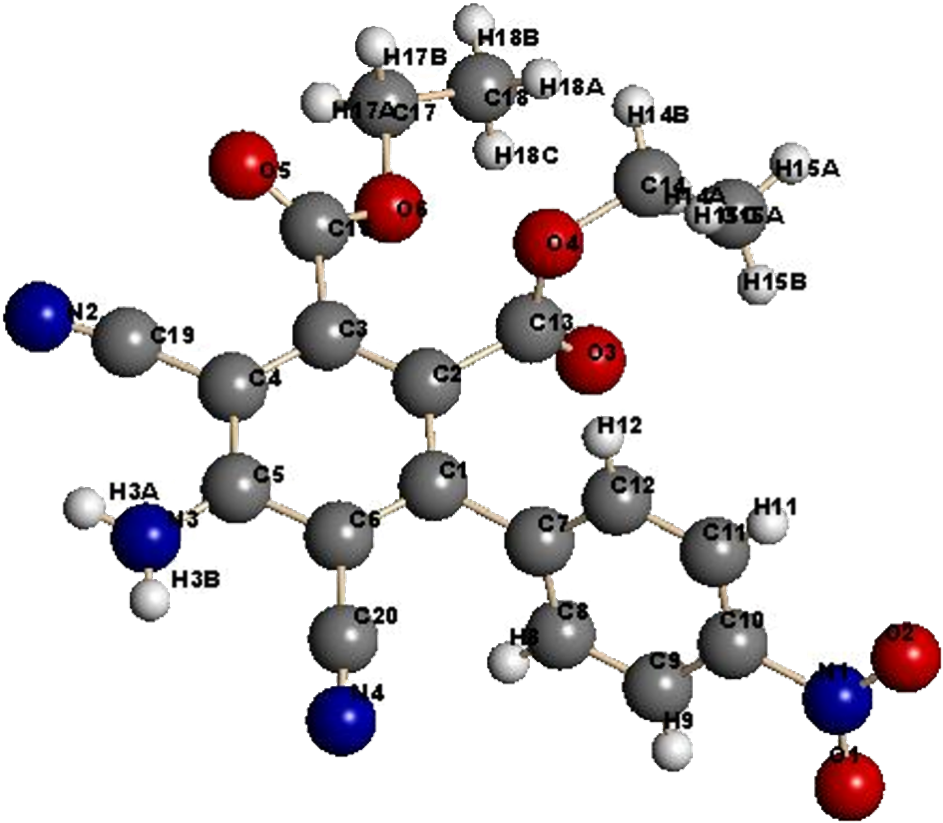
X-ray crystal structure of **37b**.

In a similar fashion, DEAD was observed to react with the *p*-nitro- and *p*-chloro-benzylidenemalononitriles **7b** and **7c** in the presence of DABCO to yield the corresponding phthalate diesters **37b** and **37c** (Figure 12).

**Scheme 11:**
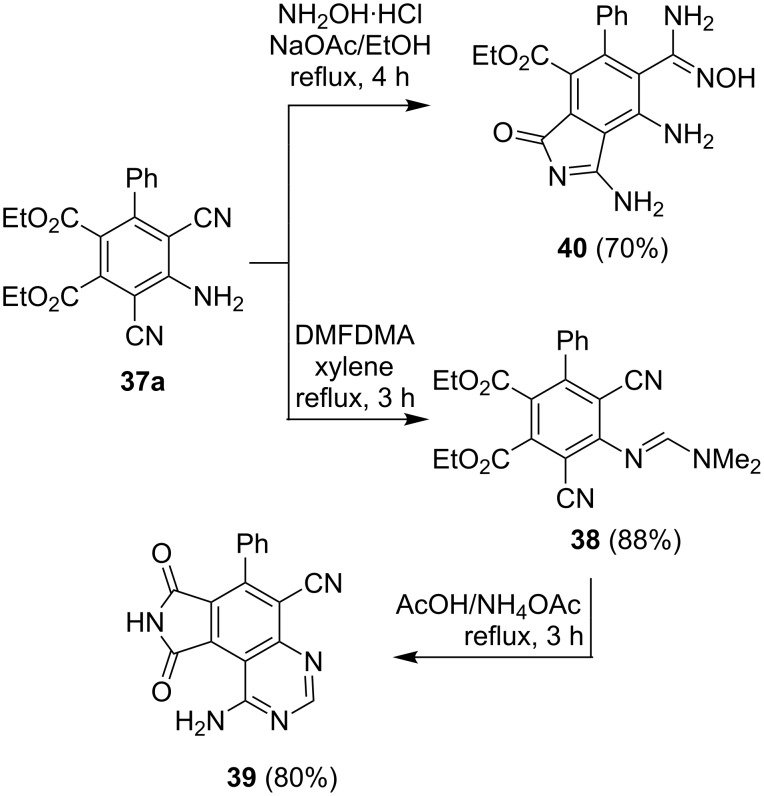
Syntheses of compounds **38–40**.

**Figure 13:**
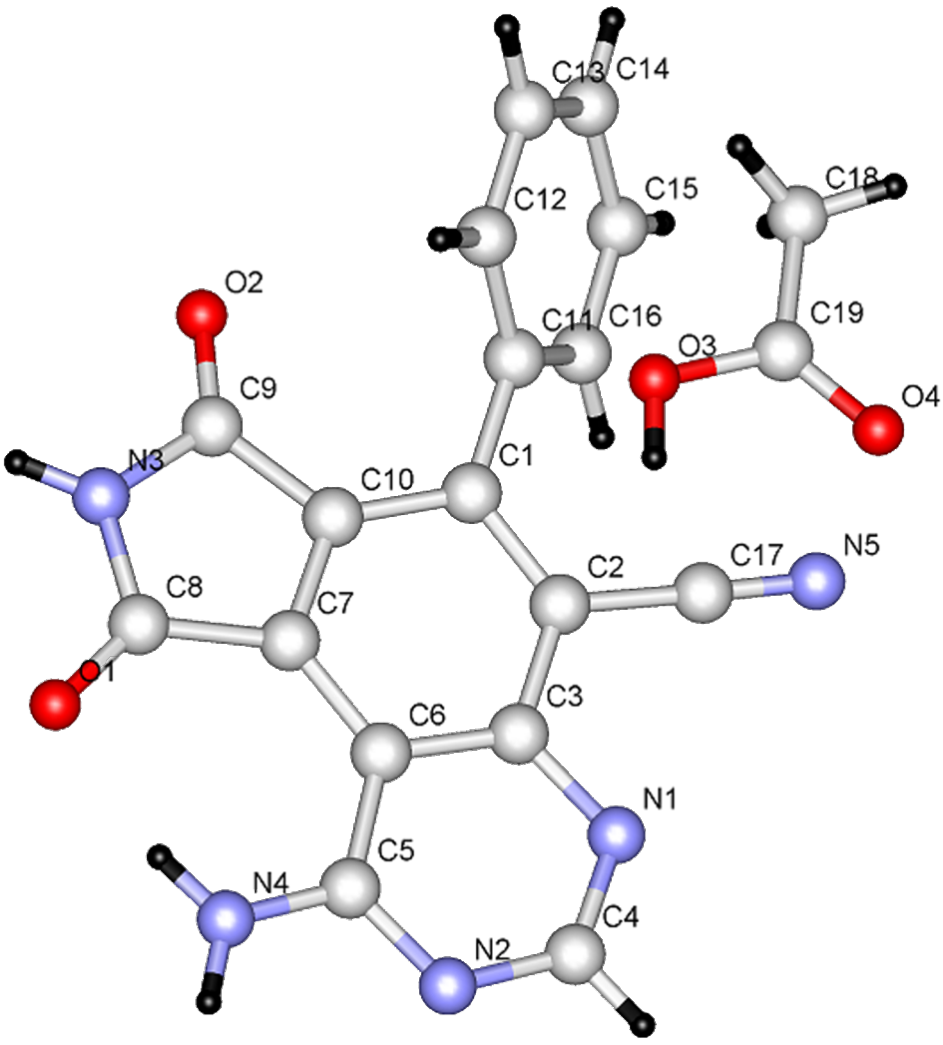
X-ray crystal structure of **39**.

A plausible mechanistic route for the formation of **37a** involves an initial addition of DABCO to DEAD to yield the intermediate zwitterionic enammonium diester **34**, which then adds to **7a** to produce zwitterion **35a**. Reaction of **35a** or its protonated form with malononitrile, which is likely generated by hydrolysis of **7a**, then forms **36a** that cyclizes and aromatizes by loss of HCN to yield **37a**. This sequence is closely related to the one described above for the formation of benzoic acid derivatives **13a** and **13b** (Scheme 6). Although they have potential utility in the field of polymer chemistry, to the best of our knowledge the preparation and chemical reactivity of only a few tetrasubstituted phthalic acid diesters have been described previously. In this effort, we observed that phthalate **37a** reacts with DMFDMA to form amidine **38**, which undergoes cyclization in refluxing AcOH/NH_4_OAc to form the 1-amino-pyrrolo[3,4-*f*]quinazoline **39**. We also found that **37a** reacts with hydroxylamine hydrochloride in ethanolic sodium acetate solution to form isoindolone derivative **40** (Scheme 11 and Figure 13).

## Conclusion

In the current investigation, we have developed new and efficient methods for the synthesis of polyfunctionalized 2-amino-4*H*-pyrans and aminobenzoic acids. In addition, we have explored the preparative potential of these substances as intermediates for the synthesis of 6-amino-5-cyanonicotinic acid derivatives **31a**,**b**, ethyl 4-amino-5*H*-pyrano[2,3-*d*]pyrimidine-6-carboxylates **33a**,**b**, 4-amino-6*H*-pyrrolo[3,4-*g*]quinazoline-9-carbonitrile **39**, and 1,7-diamino-6-(*N*'-hydroxycarbamimidoyl)-3-oxo-5-phenyl-3*H*-isoindole-4-carboxylate **40**.

## Supporting Information

File 1 Experimental.
