# Supplementary material for: Organobase-catalyzed three-component reactions for the synthesis of 4H-2-aminopyrans, condensed pyrans and polysubstituted benzenes
Source: Beilstein J Org Chem. 2014 Jan 14;10:141–9. doi: 10.3762/bjoc.10.11 (PMC3896230; doi:10.3762/bjoc.10.11)
Supplement: File 1 — Experimental. [file Beilstein_J_Org_Chem-10-141-s001.pdf]

Supporting Information

for

Organobase-catalyzed three-component reactions for the synthesis of 4*H*-2-aminopyrans, condensed pyrans and polysubstituted benzenes

Moustafa Sherief Moustafa[1], Saleh Mohammed Al-Mousawi[1,*], Maghraby Ali Selim[2], Ahmed Mohamed Mosallam[2] and Mohamed Hilmy Elnagdi[1,*]

Address: [1]Department of Chemistry, Faculty of Science; University of Kuwait, Safat, 13060, P.O. Box 5969, Kuwait and [2]Department of Chemistry, Faculty of Science at Qena, South Valley University, P.O. Box 83523, Qena, Egypt

Email: Saleh Mohammed Al-Mousawi* - salehalmousawi@hotmail.com; Mohamed Hilmy Elnagdi* - shelmi1941@yahoo.com

*Corresponding author

Experimental

General

Melting points reported are uncorrected and were determined with a Sanyo (Gallaenkamp) instrument. Infrared spectra were recorded as KBr pellets on a Jasco FTIR 6300 instrument and absorption bands are reported in cm^{-1}. ^{1}H and ^{13}C NMR spectra were determined with a Bruker DPX instrument at 400 MHz for ^{1}H NMR and 100 MHz for ^{13}C NMR as $CDCl_3$ or DMSO-d_6 solutions with TMS as internal standard. Chemical shifts are reported in δ (ppm). Mass spectra and accurate mass were measured using a GC–MS DFS Thermo spectrometer with the EI (70 eV) mode. X-ray crystallographic structure determinations were performed by using Rigaku Rapid II and Bruker X8 Prospector single crystal X-ray diffractometers. All X-ray crystal structural data can be obtained free of charge from the Cambridge Crystallographic Data Centre at www.ccdc.cam.ac.uk. All reactions were monitored by thin-layer chromatography (TLC) with ethyl acetate/petroleum ether 1:1 as the solvent and continued until all starting materials were consumed.

Synthesis of 6-amino-5-cyano-4-phenyl-4*H*-pyran-3-carboxylic acid ethyl ester) (**9**).

A mixture of ethyl propiolate (**4a**) (0.98 g, 0.01 mol) and 2-benzylidenemalononitrile (**7a**) (1.54 g, 0.01 mol) in EtOH (25 mL) in the presence of L-proline (0.23 g, 20%) was stirred at reflux for 3–4 h, cooled and then poured into ice-water. The formed solid was collected by filtration and recrystallized from EtOH to give **9** as faint yellow crystals in 85% yield; mp. 189-90 ºC. EI-HRMS: *m/z* = 270.09 (MH^+); $C_{15}H_{14}N_2O_3$ requires: *m/z* = 270.29 (MH^+); IR: 3383, 3322 (NH_2), 2195 (CN), 1706 (CO); ^{1}H NMR (400 MHz, DMSO-*d6*): δ = 1.07 (t, 3H, *J* = 8.0 Hz, CH_3), 3.97 (q, 2H, *J* = 8.0 Hz, CH_2), 4.23 (s, 1H, CH),

7.02 (br, 2H, NH_2, D_2O exchangeable), 7.16-7.34 (m, 5H, Ph-H), 7.71 (s, 1H, CH); ^{13}C NMR (100 MHz, DMSO-*d6*): δ = 164.5, 158.6, 147.7, 144.2, 133.3, 129.5 (2C), 128.4 (2C), 126.9, 111.3, 60.24, 57.3, 37.1, 13.8. MS: *m/z* (%) 270 (M^+, 50), 256 (30), 193 (80), 130 (100), 103 (70), 69 (85). CCDC 851560 contains the supplementary crystallographic data.

Synthesis of 5-amino-4,6-dicyano-biphenyl-2-carboxylic acid ethyl ester (**13a**).

A mixture of ester **9** (2.70 g, 0.01 mol) and malononitrile (0.66 g, 0.01mol) in EtOH (25 mL) in the presence of pipiridine (0.5 mL) was stirred at reflux for 3–4 h, cooled and then poured into ice-water. The formed solid was collected by filtration and recrystallized from AcOH to give **13a** as faint yellow crystals in 75% yield; mp. 185-86 ºC. EI-HRMS: *m/z* = 291.10 (MH^+); $C_{17}H_{13}N_3O_2$ requires: *m/z* = 291.31 (MH^+); IR (KBr1): 3393, 3344 (NH_2), 2237 (CN), 2225 (CN), 1700 (CO); ^1H NMR (400 MHz, DMSO-*d6*): δ = 0.85 (t, 3H, *J* = 8.0 Hz, CH_3), 3.90 (q, 2H, *J* = 8.0 Hz, CH_2), 7.25-7.28 (m, 2H, Ph-H), 7.44-7.48 (m, 5H, Ph-H, NH_2, D_2O exchangeable), 8.21 (s, 1H, CH); ^{13}C NMR (100 MHz, DMSO-*d6*): δ = 164.2, 153.8, 152.2, 140.0, 137.6, 128.5, 1280 (2C), 127.9 (2C), 118.7, 115.5, 114.8, 98.3, 95.1, 60.5, 13.3. MS: *m/z* (%) 291 (M^+, 40), 263 (15), 246 (100), 217 (10), 191 (25), 164 (30), 140 (5), 77 (5).

Synthesis of 5-amino-4'-nitro-4,6-dicyano-biphenyl-2-carboxylic acid ethyl ester (**13b**).

A mixture of ethyl propiolate (**4a**, 0.98 g, 0.01 mol) and 2-(4-nitrobenzylidene)-malononitrile (**7b**, 3.98 g, 0.02 mol) in EtOH (25 mL) in the presence of L-proline (0.23 g, 20%) was stirred at reflux for 3–4 h, cooled and then poured into ice-water. The

formed solid was collected by filtration and recrystallized from EtOH to give **13b** as yellow crystals in 60% yield; mp. 271-73 ºC. EI-HRMS: *m/z* = 336.18 (MH^+); $C_{17}H_{12}N_4O_4$ requires: *m/z* = 336.31 (MH^+); IR: 3372, 3319 (NH_2), 2224 (CN), 2211 (CN), 1700 (CO); ^{1}H NMR (400 MHz, DMSO-*d6*): δ = 1.36 (t, 3H, *J* = 8.0 Hz, CH_3), 4.36 (q, 2H, *J* = 8.0 Hz, CH_2), 7.61 (d, 2H, Ph-H), 7.65 (br, 2H, NH_2, D_2O exchangeable), 8.31 (d, 2H, Ph-H), 8.64 (s, 1H, CH); ^{13}C NMR (100 MHz, DMSO-*d6*): δ = 164.1, 153.8, 150.5, 147.4, 144.7, 140.4, 133.3 (2C), 129.5 (2C), 117.4, 115.2, 114.4, 98.0, 95.9, 61.6, 14.0. MS: *m/z* (%) 336 (M^+, 100), 308 (60), 291 (70), 275 (20), 245 (80), 217 (30), 189 (30), 163 (20), 152 (10), 83 (10), 57 (5). CCDC 876576 contains the supplementary crystallographic data.

Synthesis of 2-amino-4-(cyanomethyl)-7,7-dimethyl-5-oxo-5,6,7,8-tetrahydro-4*H*-chromene-3-carbonitrile (**18**).

A mixture of dimedone (**14**,1.40 g, 0.01 mol), 3-(piperidin-1-yl)acrylonitrile (**15**, 1.36 g, 0.01 mol) and malononitrile (0.66 g, 0.01 mol) in EtOH (25 mL) in the presence of L-proline (0.23 g, 20%) or DABCO (0.22 g, 20%) was stirred at reflux for 4 h, cooled and then poured into ice-water. The formed solid was collected by filtration and recrystallized from EtOH to give **18** as white crystals in 87% yield; mp. 232-33 ºC. EI-HRMS: *m/z* = 257.08 (MH^+); $C_{14}H_{15}N_3O_2$ requires: *m/z* = 257.11 (MH^+); IR: 3410, 3296 (NH_2), 2243 (CN), 2187 (CN), 1680 (CO); ^{1}H NMR (400 MHz, DMSO-*d6*): δ = 1.05 (s, 3H, CH_3), 1.06 (s, 3H, CH_3), 2.19-2.89 (m, 6H, CH_2, CH_2, CH_2), 3.45 (br, 1H, CH), 7.27 (br, 2H, NH_2, D_2O exchangeable); ^{13}C NMR (100 MHz, DMSO-*d6*): δ = 196.42, 164.76, 160.22, 119.04, 118.39, 109.69, 53.20, 49.90, 31.77 (2C), 28.84, 27.55, 26.07, 24.10.

MS: *m/z* (%) 257 (M^+, 4), 217 (100), 201 (10), 161 (20), 133 (10), 108 (5), 66 (5). CCDC 923274 contains the supplementary crystallographic data.

Synthesis of 7,7-dimethyl-2-(piperidin-1-yl)-7,8-dihydroquinolin-5(6*H*)-one (**21**).

A mixture of dimedone (**14**, 1.40 g, 0.01 mol) and 3-(piperidin-1-yl)acrylonitrile (**15**, 1.36 g, 0.01 mol) EtOH (25 mL) containing 5 drops of piperidine was stirred at reflux for 4 h, cooled and then poured into ice-water. The formed solid was collected by filtration and recrystallized from EtOH to give **21** as yellow crystals in 97% yield; mp 145-47 ºC; EI-HRMS: *m/z* = 258.36 (MH^+); $C_{16}H_{22}N_2O$ requires: *m/z* = 258.36 (MH^+); IR: 1653 (CO); ^{1}H-NMR (DMSO): 0.99 (s, 6H, $2CH_3$), 1.53-1.63 (m, 6H, pip-H), 2.34 (s, 2H, CH_2), 2.70 (s, 2H, CH_2), 3.67 (br, 2H, pip-H), 6.73 (d, 1H, *J* = 8, CH), 7.82 (d, 1H, *J* = 8, CH); ^{13}C-NMR (DMSO): 194.95 (CO), 162.73, 159.52, 135.24, 116.20, 104.58, 51.07, 46.11, 44.97 (2C), 32.48, 27.93 (2C), 25.29 (2C), 24.19. MS: *m/z* (%) 258 (M^+, 100), 229 (90), 215 (45), 203 (40), 175 (55), 145 (10), 119 (10), 91 (15), 84 (35). CCDC 811998 contains the supplementary crystallographic data.

Synthesis of ethyl 3-amino-4-cyano-6,6-dimethyl-8-oxo-5,6,7,8-tetrahydronaphthalene-2-carboxylate (**26**).

A mixture of dimedone (**14**, 1.40 g, 0.01 mol), ethyl propiolate (**4a**, 0.98 g, 0.01 mol) and malononitrile (0.66 g, 0.01 mol) in EtOH (25 mL) in the presence of L-proline (0.23 g, 20%) or DABCO (0.22 g, 20%) was stirred at reflux for 4 h, cooled and then poured into ice-water. The formed solid was collected by filtration and recrystallized from EtOH to give **27** as faint yellow crystals in 75% yield; mp. 255-56 ºC. EI-HRMS: *m/z* = 286.16

(MH$^+$); $C_{16}H_{18}N_2O_3$ requires: *m/z* = 286.33 (MH$^+$); IR: 3430, 3322 (NH_2), 2210 (CN), 1720 (CO), 1677 (CO); ^{1}H NMR (400 MHz, DMSO-*d6*): δ = 1.04 (s, 6H, 2CH_3), 1.33 (t, 3H, *J* = 8.0 Hz, CH_3), 2.44 (s, 2H, CH_2), 2.63 (s, 2H, CH_2), 4.34 (q, 2H, *J* = 8.0 Hz, CH_2), 7.86 (br, 2H, NH_2, D_2O exchangeable); ^{13}C NMR (100 MHz, DMSO-*d6*): δ = 194.05, 166.14, 154.99, 153.32, 134.40, 130.45, 129.28, 120.05, 115.17, 109.55, 61.20, 50.47, 33.06, 27.52 (2C), 14.07. MS: *m/z* (%) 286 (M$^+$, 100), 271 (15), 240 (40), 230 (90), 215 (50), 202 (25), 184 (50), 157 (20), 129 (30), 102 (15), 55 (15). CCDC 923273 contains the supplementary crystallographic data.

General procedure for the syntheses of **27a** and **27b**.

Independent mixtures of **9a** and **9b** (0.01 mol), each containing hydroxylamine hydrochloride (0.01 mol) and sodium acetate (0.01 mol), in EtOH (25 mL) were stirred at reflux for 3 h, cooled and then poured into ice-water. The formed solids were collected by filtration and recrystallized from EtOH to give **28a** and **28b** as white crystals.

Ethyl 5-(*N*'-hydroxycarbamimidoyl)-6-(hydroxyimino)-4-phenyl-5,6-dihydro-4*H*-pyran-3-carboxylate (**27a**).

Yield 65%; mp. 213-15 ºC. EI-HRMS: *m/z* = 319.11 (MH$^+$); $C_{15}H_{17}N_3O_5$ requires: *m/z* = 319.32 (MH$^+$); IR: 3483 (2OH), 3387, 3284 (NH_2), 1675 (CO); ^{1}H NMR (400 MHz, DMSO-*d6*): δ = 1.38 (t, 3H, *J* = 8.0 Hz, CH_3), 3.69 (q, 2H, *J* = 8.0 Hz, CH_2), 4.22 (s, 1H, CH) 5.45 (br, 2H, NH_2, D_2O exchangeable), 7.17-7.29 (m, 5H, Ph-H), 7.35 (d, *J* = 4.0 Hz, 1H, CH), 9.22 (s, 1H, OH, D_2O exchangeable), 9.30 (d, *J* = 4.0 Hz, 1H, CH), 10.08 (s, 1H, OH, D_2O exchangeable); ^{13}C NMR (100 MHz, DMSO-*d6*): δ = 166.25, 151.25,

147.52, 143.01, 142.64, 136.64, 128.35 (2C), 127.04 (2C), 126.43, 102.49, 58.99, 44.49, 14.32. MS: *m/z* (%) 319 (M^+, 50), 301 (40), 258 (60), 242 (30), 197 (55), 155 (15), 140 (25), 115 (35), 81 (55), 69 (100). CCDC 923277 contains the supplementary crystallographic data.

Ethyl 5-(*N*'-hydroxycarbamimidoyl)-6-(hydroxyimino)-2-methyl-4-phenyl-5,6-dihydro-4*H*-pyran-3-carboxylate (**27b**).

Yield 70%; mp. 249-50 ºC. EI-HRMS: *m/z* = 333.13 (MH^+); $C_{19}H_{19}N_3O_5$ requires: *m/z* = 333.34 (MH^+); IR: 3482 (OH) 3379 (OH), 3271, 3081 (NH_2), 1660 (CO); ^{1}H NMR (400 MHz, DMSO-*d6*): δ = 1.06 (t, 3H, *J* = 8.0 Hz, CH_3), 2.38 (s, 3H, CH_3), 3.91 (q, 2H, *J* = 8.0 Hz, CH_2), 4.34 (br, 1H, CH) 5.43 (br, 2H, NH_2, D_2O exchangeable), 7.16-7.25 (m, 5H, Ph-H), 8.68 (br, 1H, CH), 9.21 (s, 1H, OH, D_2O exchangeable), 9.98 (s, 1H, OH, D_2O exchangeable); ^{13}C NMR (100 MHz, DMSO-*d6*): δ = 166.95, 151.28, 147.83, 143.68, 142.66, 128.20 (2C), 127.14 (2C), 126.22, 99.35, 58.73, 44.48, 40.74, 18.98, 14.21. MS: *m/z* (%) 333 (M^+, 50), 315 (100), 296 (25), 273 (65), 257 (35), 236 (25), 211 (55), 184 (25), 168 (15), 140 (20), 128 (25), 103 (15), 77 (20).

General procedure for the syntheses of **28a** and **28b**.

Independent solutions of **28a** and **28b** (0.01 mol) in DMF (25 mL) were stirred at reflux for 4 h, cooled and then poured into ice-water. The formed solids were collected by filtration and recrystallized from EtOH to give **28a** and **28b** as white crystals.

Ethyl 6-amino-5-carbamoyl-4-phenylnicotinate (**28a**).

Yield 80%; mp. 193-95 ºC. EI-HRMS: *m/z* = 285.11 (MH^+); $C_{15}H_{15}N_3O_3$ requires: *m/z* = 285.28 (MH^+); IR: 3396, 3259 (NH_2), 3147, 3041 (NH_2), 1741 (CO), 1641 (CO); ^{1}H NMR (400 MHz, DMSO-*d6*): δ = 1.14 (t, 3H, *J* = 8.0 Hz, CH_3), 3.99 (q, 2H, *J* = 8.0 Hz, CH_2), 6.78 (br, 2H, NH_2, D_2O exchangeable), 7.17-7.38 (m, 7H, Ph-H, NH_2, D_2O exchangeable), 7.57 (s, 1H, CH). ^{13}C NMR (100 MHz, DMSO-*d6*): δ = 163.54, 144.34, 135.73, 129.57, 128.63 (2C), 127.85, 127.72 (2C), 126.79, 105.68, 83.76, 59.90, 44.29, 14.12. MS: *m/z* (%) 385 (M^+, 5), 384 (35), 256 (20), 241 (15), 213 (10), 185 (15), 171 (10), 129 (45), 111 (15), 83 (40), 73 (100).

Ethyl 6-amino-5-carbamoyl-2-methyl-4-phenylnicotinate (**28b**).

Yield 83%; mp. 252-54 ºC. EI-HRMS: *m/z* = 299.12 (MH^+); $C_{16}H_{17}N_3O_3$ requires: *m/z* = 299.33 (MH^+); IR: 3478, 3418 (NH_2), 3367, 3315 (NH_2), 1695 (CO), 1670 (CO); ^{1}H NMR (400 MHz, DMSO-*d6*): δ = 0.74 (t, 3H, *J* = 8.0 Hz, CH_3), 2.31 (s, 3H, CH_3), 3.79 (q, 2H, *J* = 8.0 Hz, CH_2), 61.5 (br, 2H, NH_2, D_2O exchangeable), 7.19-7.36 (m, 7H, Ph-H, NH_2, D_2O exchangeable); ^{13}C NMR (100 MHz, DMSO-*d6*): δ = 168.27, 168.14, 155.68, 154.05, 146.05, 137.57, 128.12 (2C), 127.73, 127.66 (2C), 117.61, 113.88, 60.15, 22.44, 13.31. MS: *m/z* (%) 299 (M^+, 100), 382 (25), 254 (20), 237 (35), 210 (10), 181 (10), 154 (10), 129 (15), 77 (5). CCDC 923276 contains the supplementary crystallographic data.

General procedure for the syntheses of **31a** and **31b**.

Independent mixtures of **9a** and **9b** (0.01 mol) containing ammonium acetate (0.25 g) in AcOH (25 mL) were stirred at reflux for 4 h, cooled and then poured into ice-water. The formed solids were collected by filtration and recrystallized from EtOH to give **31a** and **31b** as faint yellow crystals.

Ethyl 6-amino-5-cyano-4-phenylnicotinate (**31a**).

Yield 82%; mp. 178-80 ºC. EI-HRMS: *m/z* = 267.03 (MH^+); $C_{15}H_{13}N_3O_2$ requires: *m/z* = 267.10 (MH^+); IR: 3363, 3312 (NH_2), 2265 (CN), 1960 (CO); ^{1}H NMR (400 MHz, DMSO-*d6*): δ = 0.86 (t, 3H, *J* = 8.0 Hz, CH_3), 3.90 (q, 2H, *J* = 8.0 Hz, CH_2), 7.27-7.46 (m, 5H, Ph-H), 7.70 (br, 2H, NH_2, D_2O exchangeable), 8.67 (s, 1H, CH); ^{13}C NMR (100 MHz, DMSO-*d6*): δ = 164.96, 162.11, 156.69, 155.48, 137.23, 129.04, 128.44 (2C), 128.16 (2C), 115.93, 114.81, 90.95, 60.60, 13.91. MS: *m/z* (%) 267 (M^+, 35), 239 (10), 222 (100), 194 (10), 167 (10), 140 (35), 113 (10), 88 (5), 77 (20). CCDC 876575 contains the supplementary crystallographic data.

Ethyl 6-amino-5-cyano-2-methyl-4-phenylnicotinate (**31b**).

Yield 88%; mp. 234-35 ºC. EI-HRMS: *m/z* = 281.11 (MH^+); $C_{16}H_{15}N_3O_2$ requires: *m/z* = 281.31 (MH^+); IR: 3385, 3323 (NH_2), 2220 (CN), 1713 (CO); ^{1}H NMR (400 MHz, DMSO-*d6*): δ = 0.75 (t, 3H, *J* = 8.0 Hz, CH_3), 2.39 (s, 3H, CH_3), 3.84 (q, 2H, *J* = 8.0 Hz, CH_2), 7.31-7.49 (m, 7H, Ph-H, NH_2, D_2O exchangeable); ^{13}C NMR (100 MHz, DMSO-*d6*): δ = 166.84, 159.93, 159.75, 153.74, 136.18, 129.07, 128.39 (2C), 127.79 (2C), 117.50, 115.89, 87.08, 60.61, 23.23, 13.24. MS: *m/z* (%) 281 (M^+, 45), 236 (100), 209 (10), 191

(5), 167 (5), 140 (10), 77 (5). CCDC 923278 contains the supplementary crystallographic data.

Synthesis of ethyl 5-cyano-6-((dimethylamino)methyleneamino)-2-methyl-4-phenyl-4*H*-pyran-3-carboxylate (**32b**).

A mixture of **9b** (2.84 g, 0.01 mol) and dimethylformamide dimethylacetal (DMFDMA) (1.19 g, 0.01 mol) in DMF (25 mL) was stirred at reflux for 3 h, cooled and then poured into ice-water. The formed solid was collected by filtration and recrystallized from EtOH to give **33b** as yellow crystals in 81% yield; mp. 156-58 ºC. EI-HRMS: *m/z* = 339.15 (MH^+); $C_{19}H_{21}N_3O_3$ requires: *m/z* = 339.39 (MH^+); IR: 2197 (CN), 1713 (CO); ^1H NMR (400 MHz, DMSO-*d6*): δ = 1.04 (t, 3H, *J* = 8.0 Hz, CH_3), 2.38 (s, 3H, CH_3), 2.96 (s, 3H, CH_3), 3.11 (s, 3H, CH_3), 3.95 (q, 2H, *J* = 8.0 Hz, CH_2), 4.40 (s, 1H, CH), 7.17-7.34 (m, 5H, Ph-H) 8.25 (s, 1H, CH); ^{13}C NMR (100 MHz, DMSO-*d6*): δ = 165.54, 158.14, 157.60, 154.85, 144.30, 128.49 (2C), 127.46 (2C), 127.01, 119.46, 106.33, 73.27, 60.13, 40.42, 40.17, 34.21, 18.26, 13.75. MS: *m/z* (%) 339 (M^+, 35), 310 (15), 295 (5), 262 (100), 234 (35), 99 (20).

Synthesis of ethyl 4-amino-5-phenyl-5*H*-pyrano[2,3-*d*]pyrimidine-6-carboxylate (**33a**).

A mixture of **9a** (2.70 g, 0.01 mol) and dimethylformamide dimethylacetal (DMFDMA) (1.19 g, 0.01 mol) in DMF (25 mL) was stirred at reflux for 4–6 h and concentrated in vacuo. A solution of the residue in AcOH (25 mL) containing ammonium acetate (0.25 g) was stirred at reflux for 3 h, cooled and then poured into ice-water. The formed solid was collected by filtration and recrystallized from EtOH to give **34a** as white crystals in

80% yield; mp. 207 ^{o}C. EI-HRMS: *m⁄z* = 297.11 (MH^+); $C_{16}H_{15}N_3O_3$ requires: *m⁄z* = 297.11 (MH^+); IR: 3370, 3344 (NH_2), 1712 (CO); ^{1}H NMR (400 MHz, DMSO-*d6*): δ = 1.46 (t, 3H, *J* = 8.0 Hz, CH_3), 4.07 (q, 2H, *J* = 8.0 Hz, CH_2), 4.91 (s, 1H, CH), 6.94 (br, 2H, NH_2, D_2O exchangeable), 7.16-7.36 (m, 5H, Ph-H), 7.85 (s, 1H, CH), 8.08 (s, 1H, CH); ^{13}C NMR (100 MHz, DMSO-*d6*): δ = 164.68, 162.68, 161.02, 157.48, 149.01, 142.71, 128.25 (2C), 128.17 (2C), 126.96, 112.76, 95.82, 60.27, 33.80, 13.97. MS: *m/z* (%) 297 (M^+, 100), 268 (50), 252 (10), 220 (100), 192 (40), 165 (10), 140 (5), 69 (10). CCDC 923279 contains the supplementary crystallographic data.

Synthesis of ethyl 4-amino-7-methyl-5-phenyl-5*H*-pyrano[2,3-*d*]pyrimidine-6-carboxylate (**33b**).

A mixture of **32b** (3.39 g, 0.01 mol) and ammonium acetate (0.25 g) in AcOH (25 mL) was stirred at reflux for 3 h, cooled and then poured into ice-water. The formed solid was collected by filtration and recrystallized from EtOH to give **33b** as yellow crystals in 83%; mp. 152-54 ºC. EI-HRMS: *m⁄z* = 311.12 (MH^+); $C_{17}H_{17}N_3O_3$ requires: *m⁄z* = 311.34 (MH^+); IR: 3449, 3358 (NH_2), 1683 (CO); ^{1}H NMR (400 MHz, DMSO-*d6*): δ = 1.18 (t, 3H, *J* = 8.0 Hz, CH_3), 2.41 (s, 3H, CH_3), 4.05 (q, 2H, *J* = 8.0 Hz, CH_2), 4.94 (s, 1H, CH), 7.16 (br, 2H, NH_2, D_2O exchangeable), 7.18-7.33 (m, 5H, Ph-H), 8.05 (s, 1H, CH); ^{13}C NMR (100 MHz, DMSO-*d6*): δ = 165.63, 162.16, 161.03, 158.28, 156.41 143.82, 128.15 (2C), 128.06 (2C), 126.70, 108.44, 96.21, 60.21, 35.32, 18.69, 13.93. MS: *m/z* (%) 311 (M^+, 95), 282 (35), 266 (10), 234 (100), 206 (25), 188 (20), 161 (10), 137 (20), 121 (10), 95 (15), 81 (50), 69 (100).

General procedure for the syntheses of **37a**–**c**.

Independent mixtures of diethyl acetylenedicarboxylate (**4b**, 1.70 g, 0.01 mol) and arylidenemalononitriles **7a–c** (0.02 mol) in EtOH (25 mL) in the presence of 1,4-diazabicyclo[2,2,2]octane (DABCO) (0.22 g, 20%) were stirred at reflux for 3–4 h, cooled and then poured into ice-water. The formed solids were collected by filtration and recrystallized from EtOH to give **37a**–**c**.

5-Amino-4,6-dicyano-biphenyl-2,3-dicarboxylic acid diethyl ester (**37a**).

Faint yellow crystals, yield 85%; mp. 180-81 ºC. EI-HRMS: *m/z* = 363.28 (MH^+); $C_{20}H_{17}N_3O_4$ requires: *m/z* = 363.37 (MH^+); IR: 3347, 3248 (NH_2), 2229 (CN), 2227 (CN), 1747 (CO), 1730 (CO; ^{1}H NMR (400 MHz, DMSO-*d6*): δ = 0.77 (t, 3H, *J* = 8.0 Hz, CH_3), 1.28 (t, 3H, *J* = 8.0 Hz, CH_3), 3.83 (q, 2H, *J* = 8.0 Hz, CH_2), 4.32 (q, 2H, *J* = 8.0 Hz, CH_2), 7.31-7.48 (m, 5H, Ph-H), 7.52 (br, 2H, NH_2, D_2O exchangeable); ^{13}C NMR (100 MHz, DMSO-*d6*): δ = 164.6, 164.5, 153.3, 150.5, 140.6, 136.2, 129.1, 128.2 (2C), 128.0 (2C), 119.7, 114.3, 113.6, 99.2, 93.4, 62.7, 61.2, 13.5, 13.1. MS: *m/z* (%) 363 (M^+, 70), 318 (15), 290 (100), 272 (15), 245 (25), 218 (20), 191 (15), 164 (15), 152 (5), 97 (5), 57 (5). CCDC 861197 contains the supplementary crystallographic data.

5-Amino-4'-nitro-4,6-dicyano-biphenyl-2,3-dicarboxylic acid diethyl ester (**37b**).

Faint yellow crystals, yield 75%; mp. 191-93 ºC. EI-HRMS: *m/z* = 408.11 (MH^+); $C_{20}H_{16}N_4O_6$ requires: *m/z* = 408.10 (MH^+); IR: 3351, 3250 (NH_2), 2228 (CN), 2219 (CN), 1751 (CO), 1730 (CO); ^{1}H NMR (400 MHz, DMSO-*d6*): δ = 0.81 (t, 3H, *J* = 8.0 Hz, CH_3), 1.29 (t, 3H, *J* = 8.0 Hz, CH_3), 3.85 (q, 2H, *J* = 8.0 Hz, CH_2), 4.33 (q, 2H, *J* = 8.0

Hz, CH_2), 7.68 (d, 2H, Ph-H), 7.74 (br, 2H, NH_2, D_2O exchangeable), 8.35 (d, 2H, Ph-H); ^{13}C NMR (100 MHz, DMSO-*d6*): δ = 164.5, 163.8, 153.4, 149.0, 147.8, 143.3, 141.8, 129.8 (2C), 123.3 (2C), 118.0, 114.0, 113.4, 99.0, 94.2, 62.7, 61.4, 13.5, 13.1. MS: *m/z* (%) 408 (M^+, 70), 380 (10), 363 (15), 335 (100), 308 (10), 270 (10), 245 (25), 217 (30), 189 (50), 163 (15), 151 (5). CCDC 861198 contains the supplementary crystallographic data.

5-Amino-4'-chloro-4,6-dicyano-biphenyl-2,3-dicarboxylic acid diethyl ester (**37c**).
Faint yellow crystals, yield 75%; mp. 160-61 ºC. EI-HRMS: *m/z* = 397.73 (MH^+); $C_{20}H_{16}ClN_3O_4$ requires: *m/z* = 397.82 (MH^+); IR: 3338, 3211 (NH_2), 2222 (CN), 2221 (CN), 1689 (CO), 1684 (CO; ^1H NMR (400 MHz, DMSO-*d6*): δ = 0.83 (t, 3H, *J* = 8.0 Hz, CH_3), 1.28 (t, 3H, *J* = 8.0 Hz, CH_3), 3.86 (q, 2H, *J* = 8.0 Hz, CH_2), 4.34 (q, 2H, *J* = 8.0 Hz, CH_2), 7.36-7.57 (m, 4H, Ph-H), 7.61 (br, 2H, NH_2, D_2O exchangeable); ^{13}C NMR (100 MHz, DMSO-*d6*): δ = 164.5, 164.3, 153.3, 149.5, 141.1, 135.2, 134.0, 130.0 (2C), 128.3 (2C), 119.1, 114.3, 113.6, 99.3, 93.7, 62.7, 61.3, 13.5, 13.1. MS: *m/z* (%) 397 (M^+, 55), 369 (5), 352 (15), 324 (100), 306 (5), 279 (15), 252 (25), 225 (10), 217 (15), 189 (25), 162 (5), 69 (40).

Synthesis of 4,6-dicyano-5-(dimethylamino-methyleneamino)-biphenyl-2,3-dicarboxylic acid diethyl ester (**38**).
A mixture of **37a** (3.63 g, 0.01 mol) and dimethylformamide dimethylacetal (DMFDMA) (1.19 g, 0.01 mol) in DMF (25 mL) was stirred at reflux for 3 h, cooled and then poured into ice-water. The formed solid was collected by filtration and recrystallized from EtOH

to give **39** as white crystals in 88% yield; mp. 155-57 ºC. EI-HRMS: *m/z* = 418.16 (MH^+); $C_{23}H_{22}N_4O_4$ requires: *m/z* = 418.16 (MH^+); IR: 2227 (CN), 2225 (CN), 1727 (CO), 1635 (CO); ^1H NMR (400 MHz, DMSO-*d6*): δ = 0.81 (t, 3H, *J* = 8.0 Hz, CH_3), 1.28 (t, 3H, *J* = 8.0 Hz, CH_3), 3.06 (s, 3H, CH_3), 3.15 (s, 3H, CH_3), 3.89 (q, 2H, *J* = 8.0 Hz, CH_2), 4.34 (q, 2H, *J* = 8.0 Hz, CH_2), 7.35-7.51 (m, 5H, Ph-H), 8.20 (s, 1H, CH); ^{13}C NMR (100 MHz, DMSO-*d6*): δ = 164.83, 164.30, 159.74, 156.69, 149.23, 139.13, 135.86, 129.13, 128.39 (2C), 128.28 (2C), 125.37, 115.45, 114.65, 109.8, 103.39, 62.89, 61.58, 40.13, 34.26, 13.55, 13.15. MS: *m/z* (%) 418 (M^+, 35), 392 (20), 373 (15), 345 (20), 302 (20), 273 (10), 230 (15), 202 (5), 69 (10), 57 (15).

Synthesis of 1-amino-7,9-dioxo-6-phenyl-8,9-dihydro-7*H*-pyrrolo[3,4-*f*]quinazoline-5-carbonitrile (**39**).

A mixture of **38** (4.18 g, 0.01 mol) and ammonium acetate (0.25 g) in AcOH (25 mL) was stirred a reflux for 3 h, cooled and then poured into ice-water. The formed solid was collected by filtration and recrystallized from EtOH to give **39** as orange crystals in 80% yield; mp. 352-53 ºC. EI-HRMS: *m/z* = 315.07 (MH^+); $C_{17}H_9N_5O_2$ requires: *m/z* = 315.29 (MH^+); IR: 3279, 3141 (NH_2), 3098 (NH), 1770 (CO), 1735 (CO); ^1H NMR (400 MHz, DMSO-*d6*): δ = 7.52-7.56 (m, 5H, Ph-H), 8.65 (s, 1H, CH), 8.94 (br, 1H, NH, D_2O exchangeable), 12.06 (br, 2H, NH_2, D_2O exchangeable); ^{13}C NMR (100 MHz, DMSO-*d6*): δ = 172.08, 170.30, 166.36, 160.51, 159.42, 155.39, 147.19, 135.01, 133.16, 129.24 (2C), 127.94 (2C), 126.94, 116.26, 115.25, 110.37. MS: *m/z* (%) 315 (M^+, 100), 287 (10), 261 (20), 244 (5), 217 (10), 189 (20), 163 (10), 97 (10), 69 (5). CCDC 923275 contains the supplementary crystallographic data.

Synthesis of ethyl 1,7-diamino-6-(*N*'-hydroxycarbamimidoyl)-3-oxo-5-phenyl-3*H*-isoindole-4-carboxylate (**40**).

A mixture of **37a** (3.63 g, 0.01 mol) and hydroxylamine hydrochloride (0.69 g, 0.01 mol) in EtOH (25 mL) in presence of sodium acetate (0.01 mol) was stirred at reflux for 4 h, cooled and then poured into ice-water. The formed solid was collected by filtration and recrystallized from EtOH to give **40** as yellow crystals in 70% yield; mp. 268-69 ºC. EI-HRMS: *m/z* = 367.35 (MH^+); $C_{18}H_{17}N_5O_4$ requires: *m/z* = 367.36 (MH^+); IR (KBr,): 3462 (OH), 3411, 3396 (NH_2), 3365, 3334 (NH_2), 3236, 3212 (NH_2), 1736 (CO), 1713 (CO); ^{1}H NMR (400 MHz, DMSO-*d6*): δ = 0.91 (t, 3H, *J* = 8.0 Hz, CH_3), 3.94 (q, 2H, *J* = 8.0 Hz, CH_2), 6.29 (br, 2H, NH_2, D_2O exchangeable), 7.25-7.67 (m, 9H, Ph-H, 2NH_2, D_2O exchangeable), 11.27 (br, 1H, OH, D_2O exchangeable); ^{13}C NMR (100 MHz, DMSO-*d6*): δ = 170.13, 167.44, 166.94, 165.38, 143.15, 142.69, 138.96, 129.33, 129.02, 128.82 (2C), 128.15, 127.76 (2C), 118.93, 109.92, 60.81, 13.53. MS: *m/z* (%) 368 (M^+, 20), 353 (100), 336 (20), 305 (20), 290 (85), 264 (10), 219 (10), 192 (10), 164 (15), 152 (10), 57 (5).
